# Optimizing surface defect detection with YOLOv9: the role of advanced backbone models

**DOI:** 10.3389/frai.2025.1675154

**Published:** 2025-10-10

**Authors:** Zhonglin Zeng, Hongyang Wang, Chi Yao, Zile Dong, Shimin Cai

**Affiliations:** ^1^D'Amore-McKim School of Business, Northeastern University, Boston, MA, United States; ^2^School of Computer Science and Engineering, University of Electronic Science and Technology of China, Chengdu, China; ^3^Shenzhen Institute for Advanced Study, University of Electronic Science and Technology of China, Shenzhen, China

**Keywords:** defect detection, machine learning, YOLOv9, industrial quality control, neural networks

## Abstract

**Introduction:**

YOLO algorithmic models are widely utilized for detecting surface defects, offering a robust and efficient approach to identifying various flaws and imperfections on material surfaces.

**Methods:**

In this study, we explore the integration of six distinct backbone networks within the YOLOv9 framework to optimize surface defect detection in steel strips. Specifically, we improve the YOLOv9 framework by integrating six representative backbones-ResNet50, GhostNet, MobileNetV4, FasterNet, StarNet, and RepViT-and conduct a systematic evaluation on the NEU-DET dataset and the GC10-DET dataset. Using YOLOv9-C as the baseline, we compare these backbones in terms of detection accuracy, computational complexity, and model efficiency.

**Results:**

Results show that RepViT achieves the best overall performance with an mAP50 of 68.8%, F1-score of 0.65, and a balanced precision-recall profile, while GhostNet offers superior computational efficiency with only 41.2 M parameters and 190.2 GFLOPs. Further validation on YOLOv5-m confirms the consistency of the results.

**Discussion:**

The study offers practical guidance for backbone selection in surface defect detection tasks, highlighting the advantages of lightweight architectures for real-time industrial applications.

## 1 Introduction

Strip steel is a fundamental material in the manufacturing industry, and its quality is critical to ensuring product reliability and performance (Cui et al., 2025). However, due to limitations in production technology, working conditions, and equipment precision, the quality of finished strip steel products is often compromised (Kumar and Das, 2021; Afanasieva et al., 2018). Surface defects are among the most direct and observable indicators of product quality degradation. They typically refer to localized regions that deviate from the expected structure, including scratches, cracks, foreign object inclusions, contamination, and holes (Barros et al., 2025), and they exert a significant impact on product yield and operational reliability. Therefore, accurate and timely detection of such defects is crucial.

In the early stages of industrial development, traditional strip steel surface defect detection methods encompassed manual visual inspection, eddy current testing, magnetic flux leakage testing, infrared thermography, and laser scanning inspection (Chunfeng et al., 2022). Manual inspection relies on human observation, which is inherently subjective and susceptible to fatigue-related errors. Eddy current and magnetic flux leakage techniques detect defects through electromagnetic interactions, while infrared thermography and laser scanning utilize thermal patterns and high-resolution surface profiling, respectively. Despite providing foundational solutions, these approaches commonly exhibit limitations including insufficient detection accuracy, restricted inspection coverage, slow processing speed, and limited adaptability to complex defect morphologies (Zhang et al., 2022).

With the development of computer vision and machine learning technologies, related detection technologies are also evolving. Machine learning methods are widely used in automated defect recognition and classification tasks. Techniques such as Gaussian mixture model-based background subtraction with feature-driven classifiers achieved over 99% accuracy in weld defect recognition (Sun et al., 2019). Improved least squares methods combined with iterative algorithms enabled precise geometric parameter extraction for nickel-plated punched steel strip defects (Cao et al., 2020). Multi-scale local binary pattern (LBP) features, especially with Fourier-transformed histograms, improved classification accuracy when used with SVM (Liu et al., 2020). Filter-based feature selection coupled with hidden Naive Bayes classifiers enhanced robustness in steel strip defect categorization (Zhang et al., 2021). However, these methods depend heavily on handcrafted features and prior knowledge, limiting their flexibility and performance in complex defect scenarios. Therefore, the research focus has shifted toward deep learning approaches.

In recent years, deep learning has developed rapidly and has made great progress in the field of target detection. Based on the powerful learning ability and feature extraction ability of deep learning in a large amount of data, it has become the core technology of surface defect detection. A BO-CNN-BiLSTM framework, combined with a theoretical model, enhanced crown prediction accuracy and speed in hot-rolled electrical steel (Song et al., 2024). Vision transformers demonstrated a classification accuracy of 96.39% across six defect categories, offering a promising alternative to CNNs for real-time defect detection (Vasan et al., 2024). LWMS-Net, integrating Legendre multi-wavelet multi-scale theory, achieved 91.2% mAP, balancing accuracy and speed (Zheng et al., 2025). Despite the significant advancements achieved by various deep learning algorithms (Hao et al., 2022; Fu et al., 2023; Guo et al., 2025), challenges persist in handling complex and variable defect types, particularly in noisy industrial environments.

Subsequently, algorithms with higher efficiency, shorter time, higher accuracy and lower cost gradually entered people's field of vision. The algorithms mainly include single-stage and double-stage detection. The most mainstream algorithm in single-stage detection is YOLO (you only look once; Redmon et al., 2016). Recent YOLO variants emphasize different objectives. YOLOv9 introduces Programmable Gradient Information (PGI) and the GELAN backbone to preserve informative gradients and improve parameter utilization–mainly targeting accuracy on small objects under constrained compute. YOLOv10 advances end-to-end, NMS-free training via consistent dual assignments and component-level efficiency redesign, pushing the speed-accuracy frontier across scales (Wang et al., 2024b). YOLOv11 provides an engineering refresh within the Ultralytics ecosystem for detection/segmentation/OBB tasks and is widely used as a practical baseline (Khanam and Hussain, 2024). Domain-specific advances within the YOLO lineage also emphasize small-defect sensitivity and computational efficiency. Many researchers have employed YOLO and its variants to implement various surface defect detection methods for steel strips, achieving significant results (Li et al., 2018; Wu et al., 2022; Mi et al., 2023; Lu et al., 2024; Liao et al., 2025).

For instance, an improved YOLOv9-based method for steel surface inspection was proposed in the literature (Chen et al., 2025). This approach integrates depthwise separable convolution to reduce computational cost, employs a C3 module for multi-level feature fusion, incorporates BiFPN to strengthen small-target representation, and adopts DySample upsampling to preserve fine details. On their benchmark, the method reports a 1.8% improvement in mAP and a 7.4% gain in accuracy over the YOLOv9 baseline, while reducing parameters by 8.9%. These results exemplify the current trajectory of YOLO-style detectors–improving small-object detection under constrained compute–and motivate our recall-first, backbone-aware evaluation under a unified protocol in the industrial setting. These approaches effectively improve detection accuracy and real-time performance by enhancing network architectures, incorporating attention mechanisms, and integrating multi-scale feature fusion.

Based on the above, many YOLO-based surface-defect detectors still focus on claiming a universal SOTA detector or evaluate a single backbone in isolation. However, systematic and recall-oriented comparisons of modern backbones across detector frameworks under a unified industrial pipeline remain limited, especially for tiny, low-contrast, and extreme-aspect-ratio defects where conventional backbones may struggle to provide precise localization. To address this gap, this study aims to systematically evaluate the performance of YOLOv9 integrated with six different backbone networks for steel strip defect detection. The main contributions of this work are as follows:

- Six backbone networks (ResNet50, GhostNet, MobileNetV4, FasterNet, StarNet and RepViT) are embedded into the YOLOv9 framework to construct comparative models.
- Extensive experiments are conducted on the NEU-DET dataset to assess their performance in terms of precision, recall, parameter size, and computational efficiency.
- The strengths and limitations of each backbone in detecting different types of defects are analyzed, and practical recommendations are provided for backbone selection in real-world deployment scenarios.
- Determine, for a fixed industrial pipeline, which backbone-framework pair achieves the best recall-first performance at a given compute budget.
- Provide actionable guidance for backbone selection when priorities diverge (highest recall vs. best F1 vs. minimal GFLOPs/latency).

Beyond detector design, industrial inspection is tightly coupled with uncertainty propagation and reliability considerations. Recent work in Structural and Multidisciplinary Optimization (Chen et al., 2024; Zhang et al., 2018) exemplifies two pertinent directions: an enhanced Gaussian mixture model approach for nonlinear probabilistic uncertainty propagation via Gaussian splitting, which improves tractability when system responses are non-Gaussian and highly nonlinear; and an evidence-theory formulation that accommodates correlations among variables for structural reliability analysis. Together, these strands clarify how uncertainty: both aleatory and epistemic interacts with risk sensitive decisions in engineering systems. In surface defect detection, this perspective motivates our recall-first emphasis and our unified protocol for fair backbone-framework comparisons; Our benchmark provides a reproducible substrate to which such methods can be coupled for threshold calibration and risk assessment in future work.

In summary, this study focuses on enhancing YOLOv9 for steel strip surface defect detection by systematically investigating the impact of different backbone architectures. The findings offer both theoretical insights and practical guidance for selecting high-performance models in real-world industrial applications.

## 2 Materials

### 2.1 YOLOv9 baseline structure

In the pursuit of state-of-the-art real-time object detection, YOLOv9 stands out for its innovative approach to mitigating the inherent information-loss problem of deep neural networks (Wang, C.-Y. et al.). By integrating PGI (Programmable Gradient Information) with the versatile GELAN (Generalized Efficient Layer Aggregation Network) architecture, YOLOv9 not only enhances the model's learning ability but also ensures that key information is retained throughout the detection process, thereby achieving excellent accuracy and performance.

Regarding the choice of the basic architecture in this study, we select YOLOv9 rather than the more recent YOLOv10 or YOLOv11, and the reasons are as follows. Among the latest algorithms, YOLOv10 introduces one-to-one matching during training without the typical accuracy degradation and emphasizes a holistic efficiency-accuracy trade-off by redesigning components such as the head, neck, and label assignment. On the COCO benchmark, YOLOv10-S is reported to be 1.8× faster than RT-DETR-R18 at comparable AP, while YOLOv10-B achieves ~ 46% lower latency and 25% fewer parameters than YOLOv9-C at similar performance. These advantages are most pronounced in scenarios where post-processing and head overhead dominate the runtime budget. YOLOv11, in contrast, represents an engineering refresh that extends applicability across diverse tasks including detection, instance segmentation, classification, pose estimation, and oriented bounding boxes. It offers multiple model sizes (N/S/M/L/X) and a wide range of off-the-shelf checkpoints, with public documentation focusing on training and inference tooling as well as throughput-accuracy balance. In practice, YOLOv11 functions as a versatile baseline with convenient deployment support across tasks and hardware.

However, in our scenario involving high-resolution tiling, tiny and low-contrast targets, and extreme aspect ratios, the main challenge lies in feature resolution and recall rather than NMS latency. Thus, while YOLOv10's NMS-free design can improve throughput in general settings, our study prioritizes recall-first evaluation under a fixed high-resolution protocol. And then, newer families such as YOLOv10 and YOLOv11 can be seamlessly incorporated into the same unified pipeline in future work to further enrich the backbone-framework comparison.

**Figure 1:**
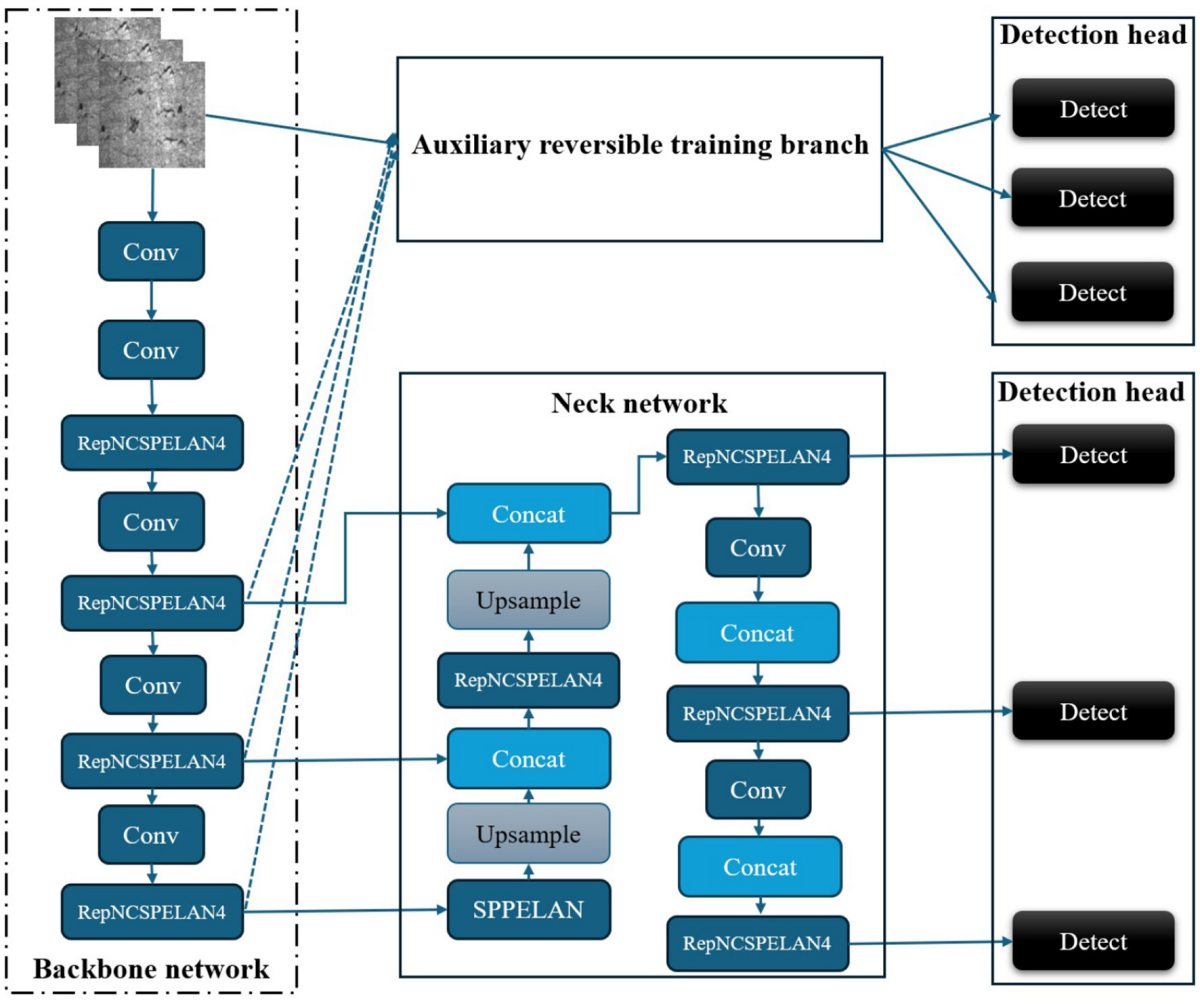
Basic structure of YOLOv9.

The architecture of YOLOv9 consists of four main components: the backbone network, the neck network, the auxiliary reversible training branch, and the detection head, as shown in Figure 1. During training, the input image is simultaneously processed by the backbone network and the auxiliary reversible training branch to ensure rich gradient flow, while in inference, the auxiliary path is discarded to improve speed. Feature fusion is performed using the RepNCSPELAN4 module, which integrates the CSPNet design and the re-parameterizable GELAN architecture. This yields a total of six multi-scale feature maps that are processed by detection heads to produce the final prediction.

Obviously, YOLOv9 demonstrates a well-balanced architecture that enhances both training dynamics and inference efficiency, laying a solid foundation for backbone network substitution and performance benchmarking.

**Figure 2:**
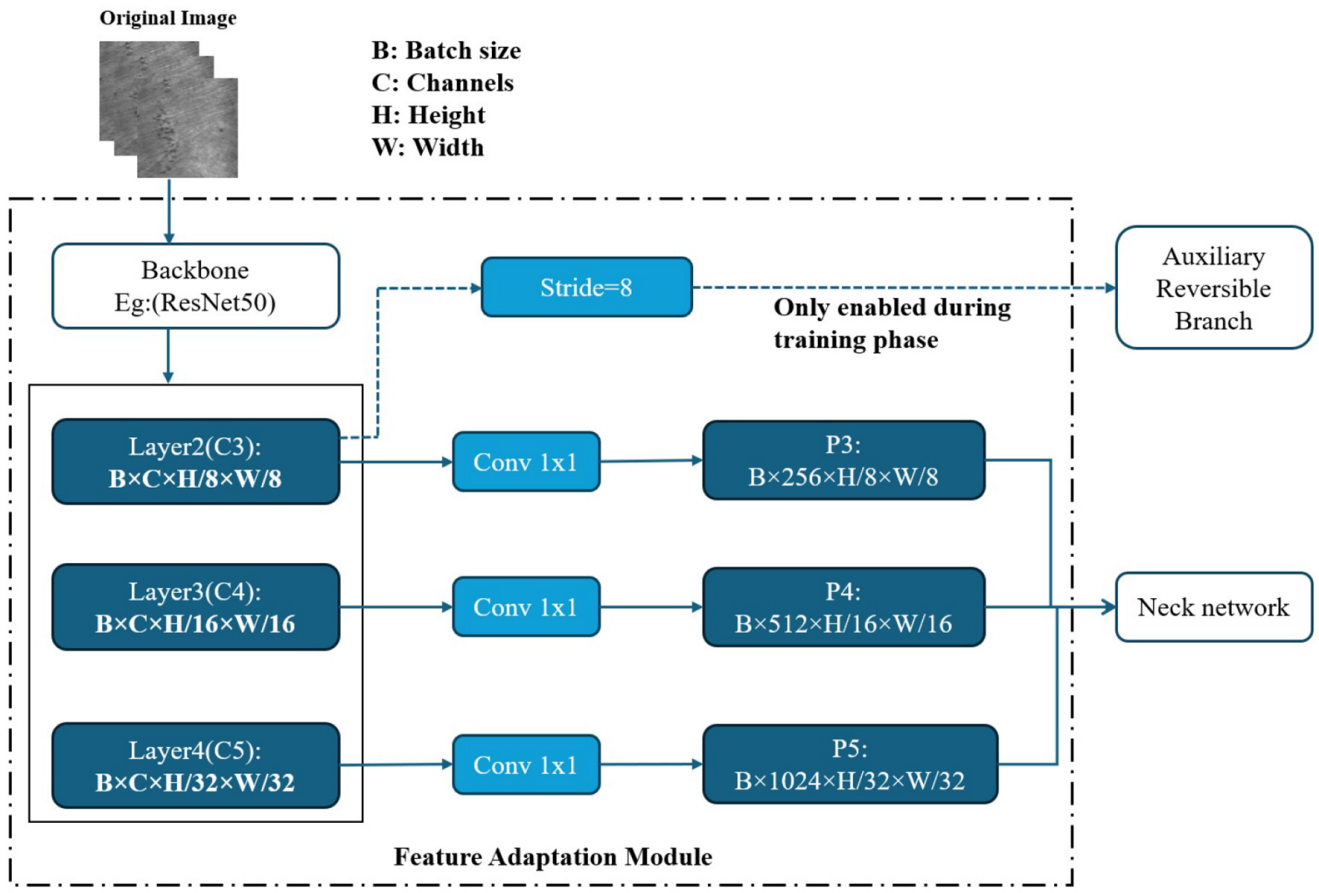
Structure of the feature adaptation module for custom backbone integration.

In the experimental section of this paper, to investigate the impact of different feature extraction capabilities, the entire GELAN backbone of YOLOv9 is replaced with six representative network architectures: ResNet50, GhostNet, MobileNetV4, FasterNet, StarNet, and RepViT. To ensure the uniformity and compatibility of the model structure, each backbone network outputs multi-scale feature maps with three different downsampling scales (8×, 16×, 32×), corresponding to the P3, P4, and P5 inputs of the Neck network in YOLOv9-C. The overall integration process of the customized backbones and the feature adaptation module is illustrated in Figure 2.

In the actual docking process, due to the difference in the number of original channels of different backbones, we introduced a 1 × 1 convolution layer after each output scale to linearly map the number of channels to unify them into the input dimension expected by YOLOv9-C Neck, that is, P3 is 256 channels, P4 is 512 channels, and P5 is 1,024 channels. The adjusted feature map is input to the Neck network to complete multi-scale feature fusion and passed to the detection head. In addition, all alternative trunks retain the output of the stride = 8 feature map (i.e., P3 layer) for connecting to the Auxiliary Reversible Branch, which is only enabled during the training phase to improve the quality of gradient propagation and model convergence speed, and is removed during the inference phase without increasing the inference overhead. In this way, we have achieved seamless replacement and unified interface of the six trunk structures under the YOLOv9-C framework, ensuring the comparability between structures and providing a basis for subsequent performance evaluation and analysis. The detailed architecture information of the corresponding adaptation layers for each backbone network is provided in Table 1.

**Table 1:** Feature adaptation configurations of different backbone networks integrated into YOLOv9-C.

**Backbone**	**Output layer**	**Downsample**	**Initial channel**	**Adjusted channel**	**Input layer**	**Method**
ResNet50	C3 / C4 / C5	8×, 16×, 32×	512 / 1024 / 2048	256 / 512 / 1024	P3 / P4 / P5	1 × 1 Conv↓
GhostNet	Stage2 / Stage3 / Stage4	8×, 16×, 32×	40 / 112 / 160	256 / 512 / 1024	P3 / P4 / P5	1 × 1 Conv↑
MobileNetV4	Stage3 / Stage4 / Stage5	8×, 16×, 32×	64 / 160 / 320	256 / 512 / 1024	P3 / P4 / P5	1 × 1 Conv↑
FasterNet	Stage2 / Stage3 / Stage4	8×, 16×, 32×	64 / 160 / 320	256 / 512 / 1024	P3 / P4 / P5	1 × 1 Conv↑
StarNet	Stage3 / Stage4 / Stage5	8×, 16×, 32×	64 / 128 / 256	256 / 512 / 1024	P3 / P4 / P5	1 × 1 Conv↑
RepViT	Stage3 / Stage4 / Stage5	8×, 16×, 32×	112 / 128 / 160	256 / 512 / 1024	P3 / P4 / P5	1 × 1 Conv↑

### 2.2 Backbone network integration

#### 2.2.1 ResNet

ResNet, originally proposed by He et al. at Microsoft Research Lab (He et al., 2016), is a classic deep convolutional network that introduces residual learning through shortcut (identity) connections to address the vanishing gradient problem in deep architectures. Unlike traditional networks where layers are stacked sequentially, ResNet enables direct gradient flow across layers by learning residual mappings.

Assuming that an input *f*(*x*) passes through the residual network and short-circuit connections are added before the second layer of the activation function, then the output becomes *f*(*x*) + *x*. In ResNet, this operation where the output is equal to the input is known as identity mapping. This operation allows the network to get the same output as the input in the worst case. The added layers do not learn anything and simply copy the features of the input, at least making the network free from degradation. The core operation is mathematically expressed as:


(1) $$\begin{array}{l} y_{l}=h\left(x_{l}\right)+F\left(x_{l}+w_{l}\right),\;x_{l+1}=f\left(y_{l}\right). \end{array}$$

Here, *x*_*l*_ and *x*_*l*+1_ denote the input and output of the *l*-th residual block, respectively. *F* represents the residual function to be learned, *h*(*x*_*l*_) = *x*_*l*_ denotes identity mapping, and *f* is the ReLU activation function. The cumulative feature learning from a shallow layer *l* to a deep layer *L* is given by:


(2) $$\begin{array}{l} x_{L}=x_{l}+\overset{L-1}{\underset{i=l}{\sum }}f\left(x_{i},w_{i}\right). \end{array}$$

In this study, ResNet50 is adopted as the backbone network within the YOLOv9 framework. Its deep residual architecture enhances feature extraction, particularly for large or complex defects, by mitigating gradient vanishing through identity mappings. The integration with YOLOv9's PGI module further aids in preserving feature information during training. Benefiting from extensive pretraining, ResNet50 improves detection accuracy across diverse defect types. However, its high computational cost, with ~ 25.6 M parameters and 4.1B FLOPs for a 224 × 224 input, along with its structural complexity, increases the risk of overfitting on limited datasets and reduces its suitability for real-time or resource-constrained applications (Han et al., 2020).

#### 2.2.2 GhostNet

GhostNet is a lightweight convolutional neural network architecture designed to reduce computational redundancy while maintaining effective feature representation. It introduces the Ghost module, which first produces a set of intrinsic feature maps via standard convolution and then generates additional “ghost" maps through inexpensive linear operations. This approach significantly reduces FLOPs and parameter count without severely impacting accuracy. The Ghost module uses the same hyperparameters (e.g., filter size, stride, padding) as standard convolution to maintain output spatial dimensions. The process is formulated as follows:


(3) $$\begin{array}{l} Y=X*f+b, \end{array}$$

where *f* is the convolution filter, *X* is the input feature map, *b* is the bias term, and *Y* is the output feature map. Then to generate the intrinsic feature map, the Ghost module uses primary convolution shown as Equation 4:


(4) $$\begin{array}{l} Y^{\prime }=X*f_{0}, \end{array}$$

where *f*_0_ is the applied filter and *Y*_0_ is the intrinsic feature map. For further feature map generation, the intrinsic feature map after primary convolution is subjected to a series of linear operations to generate the ghost feature map according to the following Equation 5:


(5) $$\begin{array}{l} y_{ij}=\Phi_{i,j}\left(y_{i}^{0}\right),\forall i=1,\ldots ,m,j=1,\ldots ,s, \end{array}$$

where $y_{i}^{0}$ is the ith intrinsic feature map in *Y*_0_, and Φ_*i, j*_ is the jth linear operation to generate the jth ghost feature map *y*_*ij*_. The last Φ_*i, s*_ is the constant mapping used to preserve the intrinsic feature map. The Equation above allows to obtain *n* = *m* × *s* feature maps. Equation 6 below represents the output of the feature map:


(6) $$\begin{array}{l} Y=\left[y_{11},y_{12},y_{13}\ldots ,y_{ms}\right]. \end{array}$$

In our implementation, GhostNet replaces the GELAN backbone in the YOLOv9 framework, while the neck and detection head remain unchanged. This substitution drastically improves inference speed and computational efficiency, making the model well-suited for low-power, real-time deployment scenarios.

However, due to its dependence on synthesized features, GhostNet may exhibit reduced sensitivity to subtle, low-contrast defects such as crazing or fine scratches. This can lead to performance degradation in fine-grained defect classification compared to more expressive architectures like RepViT (Zhang and Yuen, 2021). Nevertheless, GhostNet strikes a compelling balance between speed and accuracy, making it a practical choice for resource-constrained industrial applications.

#### 2.2.3 MobileNetV4

MobileNetV4, introduced in 2024, represents a significant advancement in lightweight neural network design by incorporating the Universal Inverted Bottleneck (UIB) (Qin et al., 2024). This unified module integrates elements from the traditional Inverted Bottleneck, ConvNeXt, and Feedforward Networks (FFN), enhancing both feature representation and structural flexibility (Sandler et al., 2018). The UIB optimizes the bottleneck design for better parameter efficiency and stronger adaptability across diverse hardware platforms.

A key component of MobileNetV4 is the depthwise separable convolution, which decomposes standard convolution into spatially focused depthwise operations and channel-wise pointwise operations. This separation reduces computational complexity while maintaining effective feature extraction, particularly for detecting fine surface anomalies. Combined with Mobile MQA attention modules and a refined Neural Architecture Search (NAS) strategy, MobileNetV4 achieves near-Pareto-optimal trade-offs between accuracy and efficiency.

In our implementation, MobileNetV4 replaces the GELAN backbone in YOLOv9 while preserving the original neck and detection head. Experimental results demonstrate improved performance in identifying surface-level defects such as scratches, pits, and corrosion, making it a compelling choice for real-time industrial inspection scenarios with limited computational resources.

#### 2.2.4 FasterNet

FasterNet addresses the inefficiency of low-FLOP lightweight networks by introducing Partial Convolution (PConv), a localized convolution strategy that significantly reduces redundant memory access and computational overhead (Chen et al., 2023). Unlike standard convolution, which operates on all input channels, PConv applies spatial convolution only to a selected subset while leaving the remaining channels unchanged (Huang and Gong, 2024). Assuming the input and output feature maps have the same size (*c, h, w*) and the kernel size is *k* × *k*, the FLOPs of standard convolution is *h* × *w* × *k*^2^ × *c*^2^, while for PConv it is reduced to $h\times w\times k^{2}\times c_{p}^{2}$, where *c*_*p*_ denotes the number of channels involved in partial convolution.

To compensate for the potential loss of feature information, a pointwise convolution (PWConv) is added after each PConv, forming an efficient feature transformation unit. FasterNet consists of four hierarchical stages, each containing multiple blocks composed of one PConv and two PWConv layers, along with embedding or merging layers for spatial downsampling and channel expansion.

When integrated as the backbone in the YOLOv9 framework, FasterNet effectively reduces inference latency and improves processing speed on edge devices. It achieves a favorable trade-off between accuracy, computational efficiency, and model complexity, particularly in surface defect detection scenarios where rapid response and limited hardware resources are essential.

#### 2.2.5 StarNet

StarNet introduces a novel nonlinear transformation mechanism known as the Star Operation, which enables implicit feature projection into high-dimensional spaces through element-wise multiplication, without relying on traditional matrix multiplication (Ma et al., 2024). Unlike conventional dot-product-based linear transformations, the star operation directly multiplies corresponding elements of two affine transformations of the input, enabling nonlinear feature interaction with minimal parameter increase. In a single-layer neural network, the star operation is defined as:


(7) $$\begin{array}{l} \left(W_{1}^{T}X+B_{1}\right)*\left(W_{2}^{T}X+B_{2}\right). \end{array}$$

For simplicity, the authors rewrite this as:


(8) $$\begin{array}{l} \left(w_{1}^{T}x\right)*\left(w_{2}^{T}x\right), \end{array}$$

where *w*_1_, *w*_2_, and *x* ∈ ℝ^(*d*+1) × 1^. This operation can be extended to multiple output channels and larger feature sets without introducing significant additional cost.

StarNet adopts a conventional hierarchical structure where spatial resolution is reduced and channel width is doubled at each stage. To meet inference efficiency requirements, batch normalization is used in place of layer normalization and is positioned after deep convolution layers, enabling fusion during deployment. Additionally, following the design principles of MobileNeXt, a depthwise convolution is appended at the end of each block to further enhance representation capability.

When integrated into the YOLOv9 framework, StarNet demonstrates strong capacity for capturing complex defect features while maintaining a lightweight design. Comparative experiments show that StarNet outperforms several compact networks such as MobileNetV3 and FasterNet in both accuracy and inference speed, highlighting its practical value for real-world surface defect detection tasks.

#### 2.2.6 RepVit

Vision Transformer (ViT) is a model proposed by Google in 2020 to directly apply the Transformer to image classification (Dosovitskiy et al., 2020). RepViT is a convolutional architecture inspired by ViT, aiming to combine the long-range modeling capability of self-attention with the computational efficiency of CNNs (Wang et al., 2024a). While ViT achieves competitive performance through global self-attention, its lack of inductive bias and high training requirements hinder its practicality on small datasets or mobile devices. To address these limitations, RepViT adopts a MetaFormer structure that separates token mixing and channel mixing, yet relies entirely on convolutional operations, making it well-suited for lightweight deployment.

Each RepViT block improves upon MobileNetV3 by decoupling spatial and channel interactions (Howard et al., 2019). Specifically, the 3 × 3 depthwise convolution is followed by a 1 × 1 convolution for channel-wise processing, and an optional Squeeze-and-Excitation (SE) module is inserted after the depthwise layer to enhance feature recalibration. To further optimize inference efficiency, RepViT employs structural reparameterization, transforming multi-branch training structures into simpler single-path equivalents during deployment. This technique reduces memory access overhead and allows the model to run with minimal latency. Experimental results demonstrate that RepViT outperforms existing lightweight ViT models across multiple vision tasks, including ImageNet classification, COCO object detection, and ADE20k semantic segmentation.

Within the YOLOv9 framework, replacing the default backbone with RepViT yields enhanced detection accuracy for both coarse and fine-grained defects, while maintaining low inference time and parameter count.

## 3 Results

### 3.1 Introduction of the dataset

In this study, both the NEU-DET hot-rolled strip steel dataset and the GC10-DET metallic surface defect dataset are employed as benchmarks to evaluate the feature extraction capabilities of different backbone networks.

**Figure 3:**
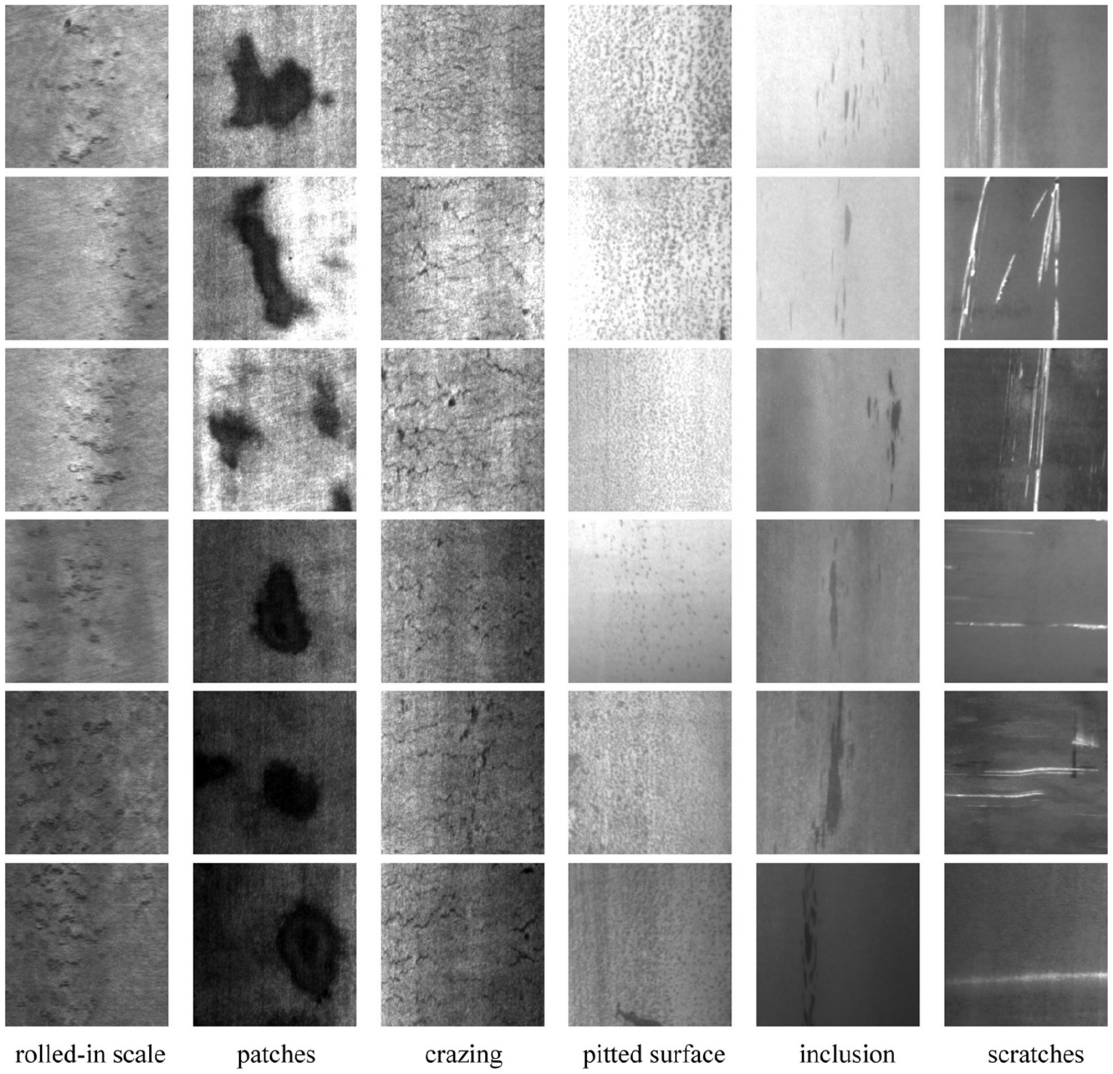
Examples of six defect categories from the NEU-DET dataset.

The NEU-DET dataset (Song and Yan, 2013), produced by Kechen Song's team at Northeastern University in China, comprises 1800 images of surface defects on hot-rolled steel strips, divided into six categories: crazing (Cr), inclusions (In), patches (Pa), pitted surface (Ps), rolled-in scale (Rs), and scratches (Sc). Each defect category contains 300 images with a resolution of 200 × 200 pixels. Considerable intra-class variability is present, as illustrated in Figure 3 (Song and Yan, 2013), where scratches may appear horizontally, vertically, or diagonally. At the same time, inter-class similarities also exist; for instance, rolled oxide, cracks, and pockmarks share overlapping features. Annotations specifying the class and location of each defect are provided by the dataset's authors, which facilitates defect detection tasks (He et al., 2019).

**Figure 4:**
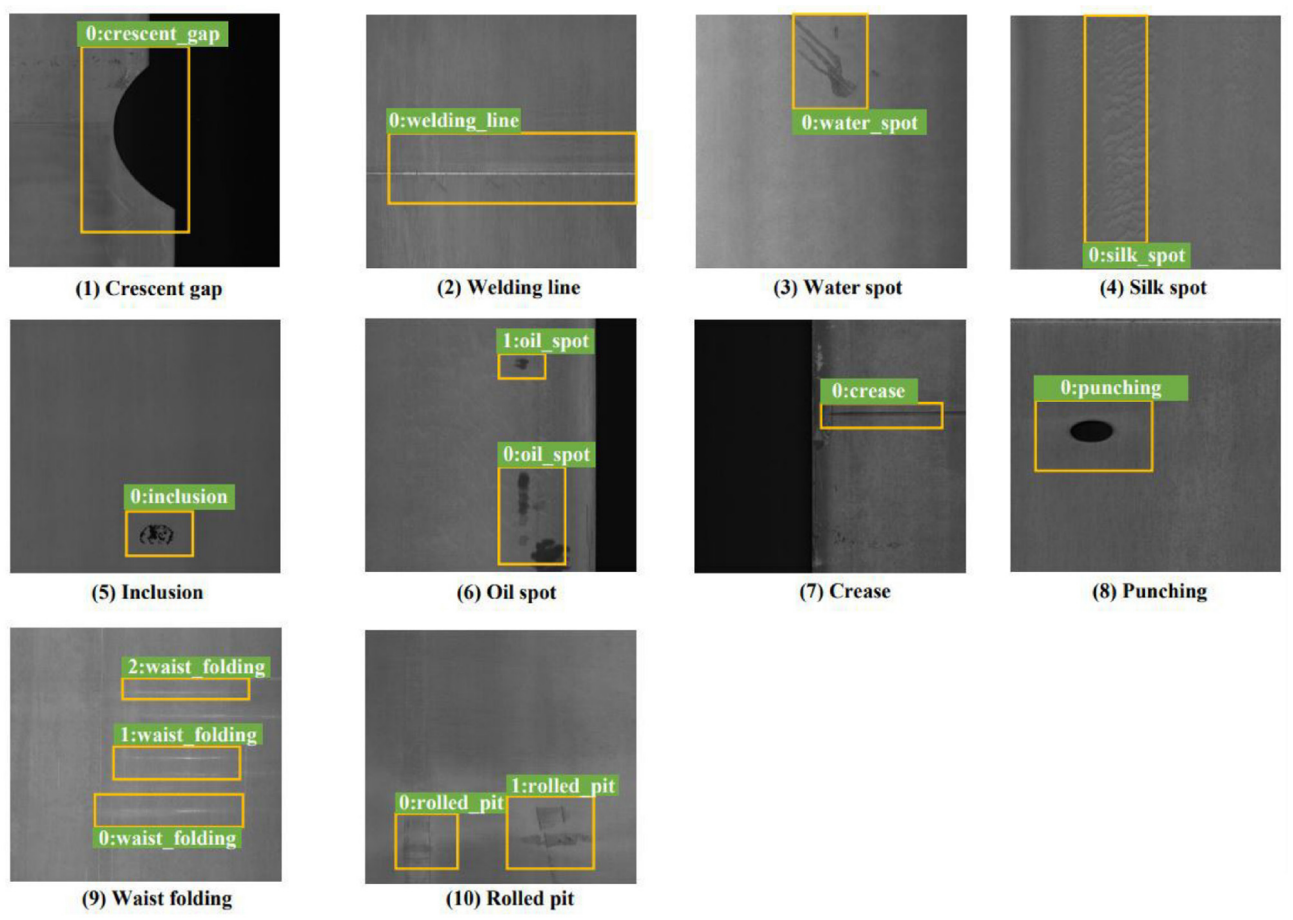
Representative samples with bounding box annotations from the GC10-DET dataset.

The GC10-DET dataset (Lv et al., 2020), released by Tianjin University in 2020, extends the evaluation to a broader range of metallic surface defect types. It consists of 2300 grayscale images with 3563 labeled objects belonging to ten categories: punched holes (Ph), welds (Wl), rescent cracks (Crg), water spots (Ws), oil spots (Os), silk spots (Ss), inclusions (In), rolling pits (Rp), creases (Cr), and waist creases (Wf). Bounding box annotations are provided for all labeled defects, and only eight images remain unlabeled. Unlike NEU-DET, GC10-DET does not include predefined train/validation/test splits, allowing flexible experimental configurations. Figure 4 illustrates representative examples with annotations. By covering a wider variety of defect patterns on steel plates, GC10-DET provides complementary evidence for assessing the robustness and generalization of detection models.

### 3.2 Environment and parameter settings

The experimental hardware configuration was Intel Core i512400F@2.5 GHz (Intel Corporation, Santa Clara, CA, USA). The processor and graphics card were NVIDIA GeForce RTX 3090, 24260MiB (Nvidia Corporation, Santa Clara, CA, USA). The software environment was CUDA11.2 and the operating system was GCC 9.4.0 on Linux. The network model was constructed based on the Python framework with Python version 3.7.12 and Pytorch version 1.13.1+cu117 shown as Table 1. For the experiments, the batch size was set to 16, the epoch was set to 100.

We adopt stochastic gradient descent (SGD) with momentum to ensure stable convergence under the high-resolution tiling regime, with an initial learning rate of 0.01 with momentum 0.937 and a 3.0 epoch warm-up to promote stable convergence under high-resolution tiling. the box regression term is upweighted (box = 7.5). For localization, we combine Distribution Focal Loss (dfl = 1.5) with an elevated box-loss gain (box = 7.5), thereby emphasizing precise bounding-box regression. The classification term is intentionally down-weighted (cls = 0.5) to mitigate overfitting to visually similar textures. The objectness term is set to obj = 0.7 to balance recall against false positives in dense scenes. For label assignment, we use a training IoU threshold of iou-t = 0.20 to enlarge the pool of positive samples for small or extreme-aspect-ratio targets. focal modulation is disabled (fl-gamma = 0.0) for stable small-object gradients. Finally, the augmentation choices emphasize robustness without excessive distortion: Mosaic is applied with high probability (mosaic=1.0) to increase small-object density and train against proposal conflicts, while MixUp is kept moderate (mixup=0.15) to avoid diluting discriminative cues. The training hyperparameters and their rationales used in all experiments are summarized in Table 2.

**Table 2:** Training hyperparameters and their rationales used consistently across YOLOv9-C and YOLOv5-m models.

**Key**	**Value**	**Rationale**
Optimizer	SGD (momentum)	Stable convergence under high-resolution tiling
lr0	0.01	Initial learning rate for rapid early progress
lrf	0.01	OneCycleLR tail; $\text{L}{\text{R}}_{\text{final}}=1\times 10^{-4}$ for precise late refinement
momentum	0.937	Damp oscillations; stabilize small-target updates
warmup_epochs	3.0	Gentle LR/momentum ramp to avoid early instability
box	7.5	Upweights box regression for accurate localization
dfl	1.5	Distribution Focal Loss strength for fine-grained box bins
obj	0.7	Recall-FP balance in dense scenes (objectness)
cls	0.5	Downweights class loss to reduce texture overfit
iou_t	0.20	Positive assignment IoU; benefits small/extreme-AR targets
fl_gamma	0.0	Disable focal modulation for stable small-object gradients
mosaic	1.0	Increases small-object density/context; trains against NMS conflicts
mixup	0.15	Moderate blending; avoids micro-texture wash-out

**Figure 5:**
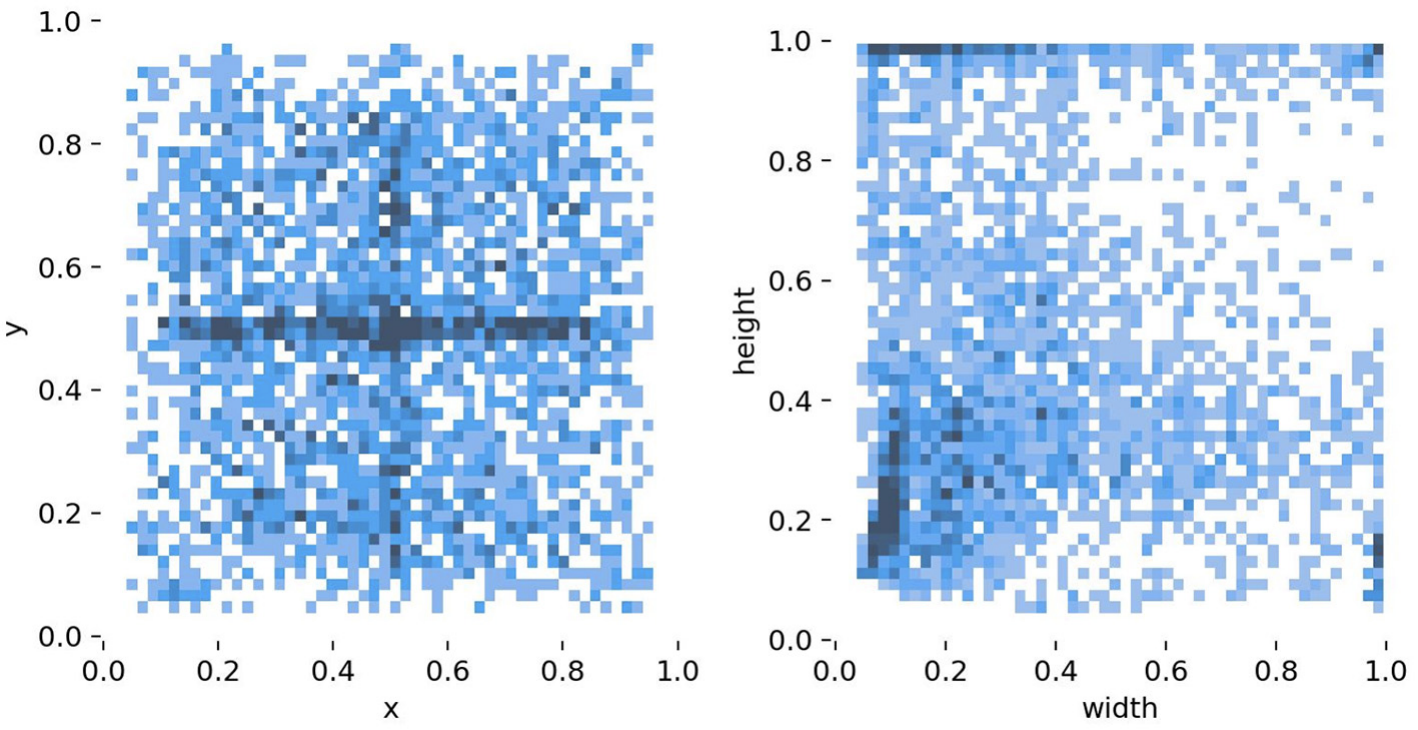
Statistical characteristics of the NEU-DET dataset, including the spatial distribution of defect centers **(left)** and the size distribution of defects **(right)**.

For the NEU-DET dataset, 1,440 images are allocated for training and 360 for validation. The dataset characteristics are illustrated in Figure 5. The left panel shows the spatial distribution of defect centers across the steel surface, where samples are unevenly distributed and exhibit a clear tendency to cluster near the midpoint of the y-axis. The right panel depicts the distribution of defect dimensions, with the horizontal and vertical axes representing width and height, respectively. The majority of defects fall into the small-scale region, and the joint distribution reveals a concentration of narrow, elongated, and irregularly shaped defects. Such properties highlight the challenges of achieving robust localization and classification, particularly for tiny or low-contrast targets. For the GC10-DET dataset, the same training-validation split ratio is applied; however, due to its larger scale, a detailed statistical analysis is not presented here.

### 3.3 Evaluation metrics

This study employs six commonly used evaluation metrics: Params, GFLOPs, mAP50, Precision, Recall, and F1-score. Among them, mAP50 (mean Average Precision at IoU=0.5) serves as the primary indicator of detection performance. It is calculated as the mean of the average precision (AP) across all categories, where each AP is derived from the area under the Precision-Recall (P-R) curve:


(9) $$\begin{array}{l} AP=\int_{0}^{1}P\left(R\right)dR,\;mAP=\frac{1}{N}\overset{N}{\underset{i=1}{\sum }}AP\left(i\right). \end{array}$$

Here, *N* denotes the total number of defect categories, and *AP*(*i*) represents the average precision of the *i*-th class.

The Precision and Recall are defined based on the number of true positives (TP), false positives (FP), and false negatives (FN). Specifically, TP refers to predicted boxes with IoU greater than 0.5 correctly matched to ground-truth boxes (each ground truth counted only once), FP refers to predicted boxes with IoU less than or equal to 0.5 or duplicate detections, and FN denotes ground-truth boxes that are not detected. Accordingly, Precision, Recall, and F1-score are computed as follows:


(10) $$\begin{array}{l} \text{Precision}=\frac{n_{\text{correct}}}{n_{\text{correctpred}}}=\frac{TP}{TP+FP}, \end{array}$$

(11) $$\begin{array}{l} \text{Recall}=\frac{n_{\text{correct}}}{n_{\text{correcttrue}}}=\frac{TP}{TP+FN}, \end{array}$$

(12) $$\begin{array}{l} \text{F1-score}=2\cdot \frac{\text{Precision}\cdot \text{Recall}}{\text{Precision}+\text{Recall}}=\frac{2TP}{2TP+FP+FN}. \end{array}$$

In addition to detection accuracy, we report model size (Params) and computational cost (GFLOPs) to evaluate inference efficiency and deployment feasibility. These metrics provide a balanced perspective on both predictive performance and resource requirements.

### 3.4 Comparative experiment

YOLOv9 supports multiple model configurations (E/C/M/S/T) to accommodate diverse application scenarios and hardware constraints. In this study, YOLOv9-C is selected as the baseline model owing to its balance between detection accuracy and computational cost. To systematically investigate the influence of different backbone architectures on model performance, we replace the default GELAN backbone in YOLOv9-C with six alternatives. Additionally, ablation experiments are conducted on YOLOv5-m to assess the consistency and generalizability of backbone behavior across detection frameworks.

**Figure 6:**
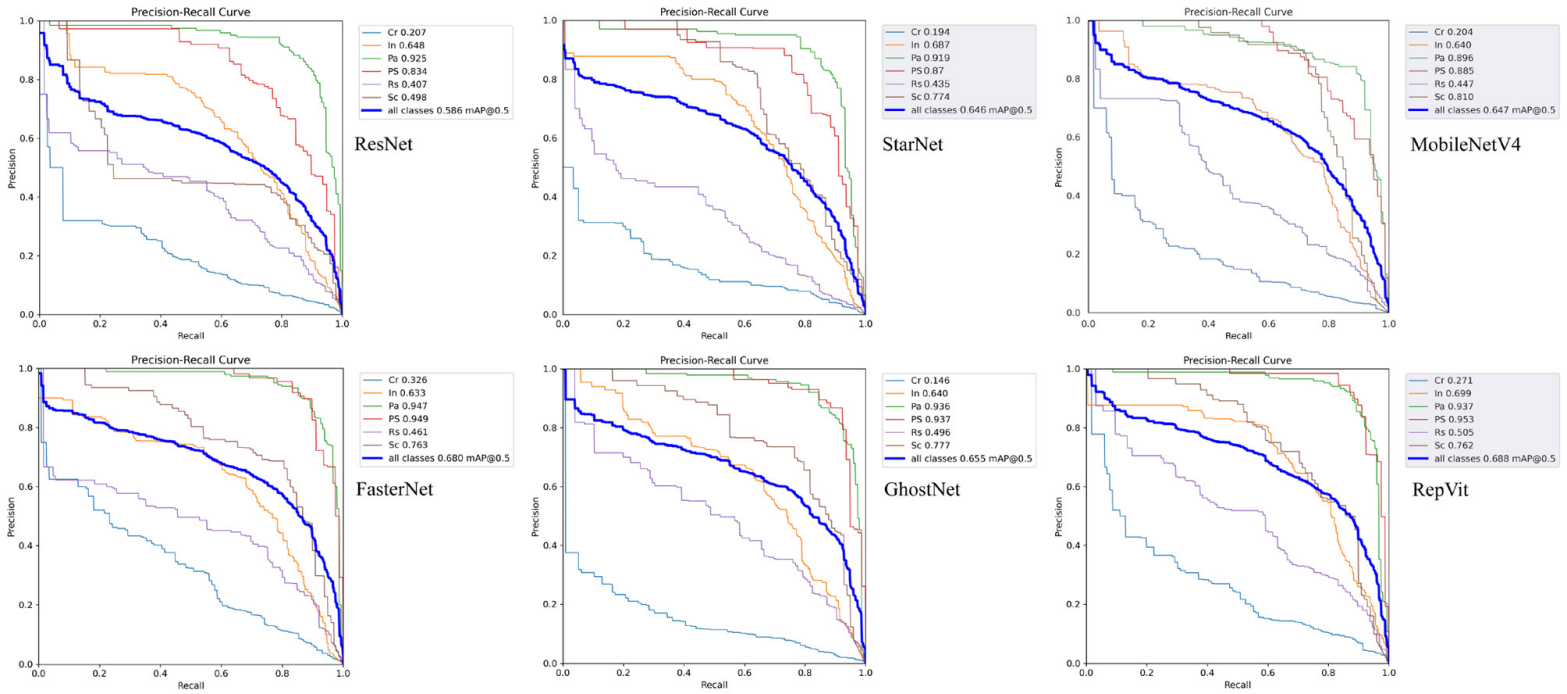
Precision-Recall curves of different backbone networks under the YOLOv9 framework: ResNet50 **(top-left)**, StarNet **(top-center)**, MobileNetV4 **(top-right)**, FasterNet **(bottom-left)**, GhostNet **(bottom-center)**, RepViT **(bottom-right)**.

Table 3 presents the experimental results of native YOLOv9-C and various backbones integrated into the YOLOv9-C framework, as well as the corresponding precision-recall (PR) curves for the six backbones are illustrated in Figure 6.

**Table 3:** Detection performance of different backbone networks integrated into the YOLOv9-C framework on the NEU-DET dataset.

**Backbone**	**Params (M)**	**GFLOPs**	**mAP50 (%)**	**Precision (%)**	**Recall (%)**	**F1-score**
Native YOLOv9-C	50.7	236.7	58.2	53.1	59.3	0.56
ResNet50	68.9	266.6	58.6	55.8	59.7	0.58
GhostNet	**41.2**	**190.2**	65.5	61.0	62.4	0.62
Mobilenetv4	45.3	207.9	64.7	**73.5**	59.6	0.65
FasterNet	43.7	196.2	68.0	58.8	**70.5**	0.64
StarNet	41.4	191.3	64.6	62.3	61.1	0.62
RepViT	46.3	205.0	**68.8**	61.8	68.6	**0.65**

Bold values indicate the best-performing model for each metric (highest value in the column).

#### 3.4.1 Baseline model analysis

We adopt ResNet50 as the baseline backbone, considering its widespread usage and solid performance in visual recognition tasks. This allows for a reasonable and consistent comparison when evaluating other backbone networks within the YOLOv9-C framework in terms of detection accuracy, computational efficiency, and model size.

On the NEU-DET dataset as shown in Table 3, the native YOLOv9-C model uses 50.7 million parameters and 236.7 GFLOPs, whereas the ResNet50 variant uses 68.9 million parameters and 266.6 GFLOPs which is the highest value among the six backbone networks, representing increases of 18.2 million parameters and 29.9 GFLOPs. These additional resources yield modest accuracy gains: mAP50 rises from 58.2% to 58.6% , precision from 53.1% to 55.8%, recall from 59.3% to 59.7%, and the F1-score from 0.56 to 0.58.

Specifically, ResNet50 achieves an mAP50 of 58.6%, indicating a moderate average accuracy. Its precision is 55.8%, suggesting limited capability in distinguishing defective from normal samples. The recall rate reaches 59.7%, 3.9% higher than the precision, showing relatively stronger performance in minimizing missed detections–albeit at the cost of increased false positives. This imbalance leads to an F1 score of 0.58, the lowest among all compared backbones.

From the perspective of architectural characteristics, ResNet50 benefits from residual connections that mitigate gradient vanishing and facilitate the extraction of deep semantic features. However, such high-level abstraction often compromises sensitivity to fine-grained surface anomalies like scratches or pits. Specifically, the bottleneck structure with 1 × 1 convolutions tends to reduce channel dimensionality, resulting in the loss of critical shallow details–particularly detrimental for pixel-level defect detection.

As shown in the precision-recall curves in Figure 6, ResNet50 performs well in detecting Pa and Ps defects, with mAP50 scores of 92.5% and 83.4%, respectively. However, the network has limited feature extraction for Rs and Sc defects, with mAP50 of only 40.7% and 49.8%, respectively. In particular, the network is very weak for Cr defects, with a mAP50 of only 20.7%, which shows that the Resnet50 network is very good at extracting features in the form of points and lumps, and then has limited ability to extract features in the form of long, thin, faint and inconspicuous defects.

#### 3.4.2 Improved backbone analysis

To enhance detection performance and computational efficiency, several lightweight or hybrid-structured backbones were evaluated as replacements for ResNet50 referred to Figure 5.

As a backbone network, GhostNet shows a significant balance between lightweight and detection performance. Its parameter volume is 41.2 M, ranking second lightest among the six compared networks, only slightly higher than StarNet's 41.4 M, but 0.398 less than the traditional residual network ResNet50. In terms of computational complexity, GhostNet's floating-point operations are 190.2 GFLOPs, which is 0.286 lower than ResNet50, and its computational efficiency is second only to StarNet. This advantage is due to GhostNet's unique feature generation mechanism, which generates “phantom features" by splitting the original feature channels and applying linear transformations, which greatly reduces redundant calculations while retaining key information.

In the detection performance indicators, GhostNet's mAP50 reached 65.5%, significantly better than ResNet50 and Mobilenetv4, indicating that it has a strong ability to locate multi-scale defects. However, its classification precision of 61.0% is lower than that of Mobilenetv4 of 73.5%, which may be due to the noise interference introduced by the phantom features in the uniform background. The recall rate of 62.4% and F1-score of 0.62 are at the middle level, reflecting its compromise optimization between missed detection and false detection. Compared with the best-performing RepViT, GhostNet has a gap in absolute accuracy, but its parameter volume and computational complexity are reduced by 0.11 and 0.078 respectively, making it more suitable for industrial scenarios with limited computing power. In addition, GhostNet's recall rate is better than StarNet, indicating that its defect coverage is more robust.

However, its capability in extracting features for Cr defects is notably weak, with a mAP50 of just 0.146. Similarly, its ability to extract features for Sc defects is also reduced, with a mAP50 of 77.7%. These results indicate that while GhostNet holds a distinct advantage in computational speed and parameter efficiency, it compromises some detection capabilities in return.

MobileNetv4 exhibits the significant characteristics of “high precision but limited recall" in the task of strip surface defect detection. Its parameter volume is 45.3M and its computational workload is 207.9 GFLOPs. Although it is significantly lighter than ResNet50, it has not reached the extreme compression level of GhostNet. The network ranks first among the six backbone networks with an precision of 73.5%, an increase of 0.112 over the second-ranked StarNet, indicating that it has unique advantages in distinguishing real defects from background noise. However, its recall rate of 59.6% ranked last, revealing a serious problem of missed detection, which may be related to the excessive compression of shallow feature resolution by its deep separable convolution. In terms of comprehensive detection performance, the mAP50 of MobileNetv4 is 64.7%, slightly lower than the similar lightweight networks FasterNet and GhostNet, reflecting that its multi-scale defect localization capability has a bottleneck. Meanwhile, It balances feature extraction for Pa, Ps, and Sc defects with mAP50 scores of 89.6%, 88.5%, and 81.0%, respectively, performing comparably to the Resnet50 network in extracting features for Cr defects, albeit the mAP remains low at 20.4%. However, due to the lack of recall rate and comprehensive performance, it is necessary to optimize the feature retention strategy and loss function design.

Conversely, the FasterNet network exhibits the best overall recall performance among the networks, recording a rate of 70.5%. It shows marked improvements in defect feature extraction, particularly for Cr defects, where it achieves a mAP50 of 32.6%, an approximate 0.12 increase over networks like Resnet50. The performance in extracting features for Pa and Ps defects has also slightly increased by 0.07% and 8%, respectively. However, its ability to extract features for In and Rs defects remains weak, with mAP50 scores decreasing by ~ 3% for In and 1.2% for Rs.

The performance of the StarNet network is mediocre in terms of parameter count, possessing 0.2 M more than GhostNet. However, this increase in parameters does not translate into better defect feature extraction capabilities, as its overall mAP50 remains 0.7% lower than that of GhostNet.

**Figure 7:**
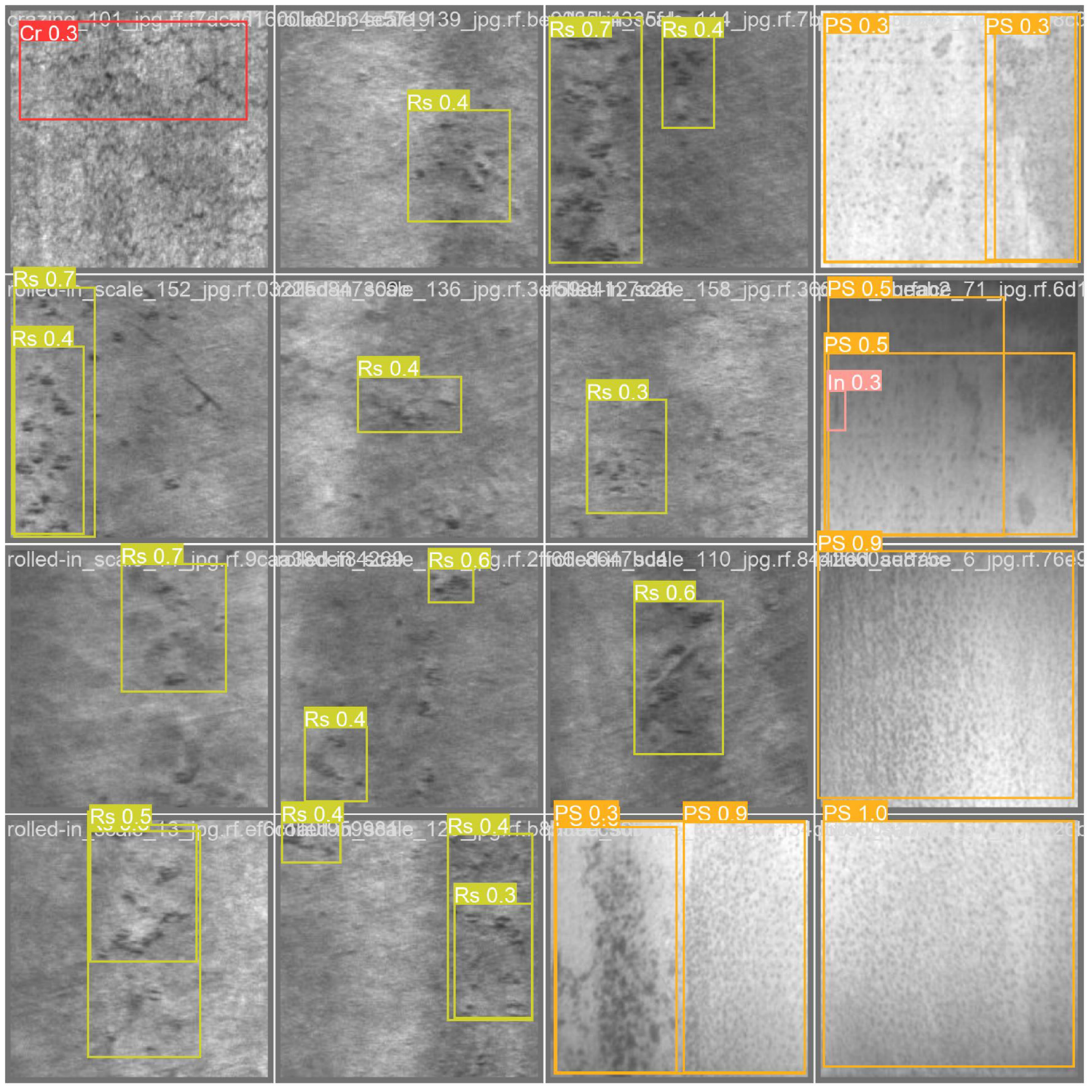
The visualization of object detection results using the RepViT backbone integrated into the YOLOv9-C framework.

RepViT showed a leading overall performance in the task. The architecture is based on the visual Transformer design and ranks first among the six backbone networks with an mAP50 of 68.8%, 0.8% higher than the second-place FasterNet. In particular, it achieved AP values of 69.9% and 50.5% in the detection of In defects and Rs defects, respectively, verifying its ability to characterize multi-scale texture features. Its recall rate of 68.6% and F1 score of 0.65 also ranked first, indicating that it has significant advantages in missed detection control and precision-recall balance. This performance improvement stems from RepViT's hybrid attention-convolution collaborative mechanism: local window self-attention is used to capture cross-regional semantic associations, while lightweight convolution is used to retain detailed information of microscopic defects. It is worth noting that although RepViT's parameter count and computational complexity are slightly higher than GhostNet, its optimized design for industrial defect characteristics makes the computing resource input-output ratio significantly better than that of traditional networks. This network provides the best solution for high-complexity defect detection scenarios. Here, we present the detection results of RepViT as shown in Figure 7.

#### 3.4.3 Detection limitations

Although improved backbone architectures enhance detection performance, limitations in detecting certain defect types are analyzed in this subsection. In particular, detection performance for Cr and Rs is suboptimal across all six backbone architectures evaluated. As depicted in Figure 5, the center heatmap shows a strong spatial prior: defects cluster in a horizontal band around the image midline (*y* = 0.5), likely induced by the acquisition setup. This crowding increases the likelihood of proposal overlap and NMS collisions, particularly for elongated instances such as Cr and Rs, and may also encourage positional overfitting with reduced confidence at top or bottom margins. the width-height distribution indicates that small objects dominate, and a sizeable fraction exhibits extreme aspect ratios. Such geometry makes the IoU highly sensitive to tiny localization errors, leading to “near-miss” failures around common mAP thresholds. These targets rely heavily on high-resolution, shallow features; insufficient input resolution or overly aggressive augmentations can attenuate micro-textures and further depress recall. Consistent with these properties, crazing and rolled-in scale –typically thin, long, and low-contrast–shows inferior detection performance relative to other classes. Thus, we decompose Cr errors into the following categories:

- Near-miss IoU (0.4-0.5),
- Score-threshold Misses,
- NMS Collisions.

The above content explains why Cr AP is low under a baseline backbone where ResNet50 mAP50 for Cr of 20.7% in our analysis and why a recall-oriented backbone (FasterNet) improves Cr of 32.6%.

### 3.5 Cross-dataset validation on GC10-DET

To further assess the generalization ability of the proposed backbones and YOLOv9 framework, we perform cross-dataset validation on the GC10-DET benchmark. The training and evaluation protocols are kept consistent with those used for NEU-DET to ensure a fair comparison.

The detection results on GC10-DET are summarized in Table 4, providing additional evidence on the robustness of backbone-specific performance trends across datasets.

**Table 4:** Detection performance comparison of various backbone networks integrated into the YOLOv9-C framework on the GC10-DET dataset.

**Backbone**	**Params (M)**	**GFLOPs**	**mAP50 (%)**	**Precision (%)**	**Recall (%)**	**F1-score**
Native YOLOv9-C	50.7	236.7	49.9	**67.1**	46.8	0.55
ResNet50	69.0	266.7	51.0	49.1	55.8	0.52
GhostNet	**41.2**	**190.3**	51.8	45.8	56.1	0.50
Mobilenetv4	45.4	208.0	49.5	54.3	51.8	0.53
FasterNet	43.7	196.3	54.4	56.6	55.6	0.56
StarNet	43.9	197.1	55.4	54.3	56.4	0.55
RepViT	46.4	205.0	**56.0**	60.4	**57.1**	**0.58**

Bold values indicate the best-performing model for each metric (highest value in the column).

As a framework reference, the native YOLOv9-C Baseline achieves 49.9% mAP50, with the highest precision of 67.1%, 46.8% recall, and 0.55 F1-score with 50.7 million parameters and 236.7 GFLOPs. Exhibiting a precision-oriented profile with the lowest recall among all configurations.

We again adopt ResNet50 as the baseline backbone due to its wide usage and stable optimization behavior. On GC10-DET, ResNet50 contains 69.0 M parameters and requires 266.7 GFLOPs–the largest compute among the compared models. It attains mAP50 of 51.0%, Precision of 49.1%, Recall of 55.8%, and F1 of 0.52. The recall-precision gap is 0.07, 0.07, 0.07 respectively indicating a tendency to reduce missed detections at the expense of more false positives. While residual connections aid deep semantic extraction, the bottleneck design with 1 × 1 compression can attenuate shallow textures, yielding moderate accuracy and F1-score on GC10-DET. With 41.2 M parameters and 190.3 GFLOPs, GhostNet is among the most efficient options. It achieves mAP50 of 51.8%, Precision of 45.8%, Recall of 56.1%, and F1-score of 0.50. The results confirm GhostNet's suitability under compute constraints, while the precision shortfall relative to heavier or hybrid designs mirrors its behavior on NEU-DET.

MobileNetV4 with 45.4 M parameters, 208.0 GFLOPs yields mAP50 of 49.5%, Precision of 54.3%, Recall of 51.8%, and F1-score of 0.53. Basically consistent with NEU-DET, it remains precision-leaning with comparatively lower recall, suggesting that aggressive depthwise separable compression can limit sensitivity to small or low-contrast instances. FasterNet provides balanced performance with mAP50 of 54.4%, Precision of 56.6%, Recall of 55.6%, and F1-score of 0.56 with 43.7 M parameters and 196.3 GFLOPs. Although it does not top recall on GC10-DET, it remains competitive across metrics and continues to be a strong choice when minimizing missed detections under moderate compute. StarNet offers a well-balanced accuracy-efficiency point, slightly outperforming GhostNet in mAP50 while preserving comparable computational cost. RepViT delivers the strongest all-around results on GC10-DET with mAP50 of 56.0%, Precision of 60.4%, Recall of 57.1%, and F1-score of 0.58. Relative to the baseline, it improves mAP50 and F1-score by 5%, 0.06 respectively and also achieving the highest recall among the six backbones, indicating better discrimination and coverage on this dataset.

The analysis reveals that three categories: Cr, In, and Rp, consistently exhibit low detection performance across all examined backbones. Validation results obtained from ResNet50, StarNet, GhostNet, MobileNetV4, FasterNet, and RepViT confirm this pattern. Specifically, the per-class mean average precision at IoU 0.50 remains within the range of 0.13 to 0.19 for Cr, 0.12 to 0.16 for In, and 0.12 to 0.20 for Rp. Correspondingly, recall values are also limited, with Cr ranging from 0.125 to 0.25, In from 0.082 to 0.205, and Rp from 0.069 to 0.135. These results demonstrate that, regardless of backbone design, the detection of these particular defect types remains recall-limited and highlights their inherent difficulty within the dataset.

Cr contains few instances (16 in our split) and typically appears as thin, elongated, low-contrast traces. Such extreme aspect ratios make IoU highly sensitive to sub-pixel localization errors, producing near-miss failures around common thresholds; the crowded ridge-like patterns also induce NMS suppression when multiple narrow proposals overlap. Precision remains low (21.4%–37.8%) and recall is uniformly poor (12.5%–25.0%) across backbones, indicating simultaneous challenges in both scoring and localization for weak-signal, scarce examples. Inclusions are minute, speck-like targets with very small bounding boxes and weak contrast against textured steel surfaces. They are easily confounded with benign spots or background artifacts, leading to score-threshold misses and assignment instability at small strides. Correspondingly, recall remains low (8.2%–20.5%) even when precision is moderate, reflecting a heavy reliance on high-resolution shallow features. Rp instances are sparse (29 in our split) and often manifest as quasi-linear/elongated textures that are visually proximate to scratches or other linear defects. Detectors tend to fire only on the most salient cases. e.g., MobileNetV4 shows high precision of 91.3%, but extremely low recall of 6.9% which is a signature of threshold sensitivity and NMS collisions along linear structures.

Across GC10-DET, Cr, In, Rp are dominated by the same failure modes identified on NEU-DET: near miss IoU, score threshold misses, and NMS collisions arising from small scale, extreme aspect ratios, low local contrast, and class sparsity. These effects are consistent across backbones, underscoring the need for recall-oriented designs and strong high-resolution features when these categories are operationally critical.

### 3.6 Cross-framework ablation validation

To verify that our conclusions are not limited to a single detector family, we integrate the six backbones into the YOLOv5-m framework and evaluate them on the same NEU-DET dataset using identical metrics, as summarized in Table 5.

**Table 5:** Detection performance of different backbone networks into the YOLOv5-m framework on the NEU-DET dataset.

**Backbone**	**Params (M)**	**GFLOPs**	**mAP50 (%)**	**Precision (%)**	**Recall (%)**	**F1-score**
ResNet50	27.5	72.1	33.3	34.5	41.0	0.37
GhostNet	**1.03**	**2.1**	55.0	64.8	53.9	0.59
Mobilenetv4	4.72	19.0	55.8	**69.1**	46.2	0.55
FasterNet	11.6	22.6	62.5	59.1	**63.7**	0.61
StarNet	9.47	18	61.2	73.6	52.2	0.61
RepViT	5.75	15.7	54.2	50.1	57.3	0.53

Bold values indicate the best-performing model for each metric (highest value in the column).

The cross-framework results largely preserve the backbone-specific tendencies observed under YOLOv9-C while also revealing the impact of the detector framework on the recall/compute trade-off. Under YOLOv5-m, ResNet50 exhibits a pronounced performance drop where mAP50 = 33.3%, F1-score = 0.37, underscoring its inefficiency in a lightweight configuration. In contrast, FasterNet and StarNet retain strong overall balance with F1-score of 0.61, while FasterNet achieving the highest Recall of 63.7% among the six backbones. GhostNet, despite having only 1.03 M parameters and 2.1 GFLOPs, delivers mAP50 of 55.0% and Precision of 64.8%, highlighting its suitability for low-resource deployment. MobileNetV4 maintains the expected precision-dominant profile with 69.1% precision, and 46.2% recall, mirroring its behavior under YOLOv9-C. RepViT attains mAP50 of 54.2% and F1 of 0.53, with stable behavior across frameworks. Together, these results indicate that while absolute numbers shift, backbone-level characteristics remain consistent across frameworks. This cross-framework ablation demonstrates that our observations are not artifacts of a single YOLO implementation. Specifically:

- Backbones favoring recall (e.g., FasterNet) continue to minimize missed detections;
- Precision-first behavior of MobileNetV4 persists across frameworks;
- Ultra-efficient GhostNet provides a competitive accuracy-efficiency trade-off in constrained settings.

The comparison thus supports our central claim that framework and backbone jointly determine the recall/compute trade-off, rather than backbone alone. Overall, the comparison supports the central claim that both the detector framework and the backbone jointly determine the recall-compute trade-off, rather than the backbone alone.

## 4 Discussion

Beyond aggregate numerical comparisons, this study provides deeper insight into how different backbone architectures influence the detection of various defect types. Models equipped with attention mechanisms or hybrid designs, such as RepViT, demonstrate superior capacity in capturing complex spatial patterns and fine-grained defect features, particularly under visually cluttered conditions. Meanwhile, lightweight backbones like GhostNet offer an effective compromise between accuracy and computational efficiency, making them appealing for latency-sensitive applications. Per-class analyses further reveal that certain challenging defect categories–such as Cr, In, Rp, and Rs remain consistently recall-limited, regardless of overall model performance. This highlights the importance of recall-oriented designs and high-resolution feature representation, especially in safety-critical industrial inspection scenarios where missing a defect may incur substantial risk.

These insights underscore a central message: backbone selection should not be guided solely by conventional metrics such as speed or precision. Instead, it must also account for sensitivity to defect characteristics and the practical constraints of the deployment environment. For example, applications that demand high throughput may prioritize latency-optimized models, whereas those involving rare or safety-critical defects may require architectures that favor recall and fine-detail sensitivity. Our results provide practical guidance in this context, demonstrating that different backbones present trade-offs that are not universally optimal but context-dependent. Notably, the consistency of trends across both YOLOv9-C and YOLOv5-m frameworks lends robustness to these observations, indicating that the underlying design principles generalize across detection heads and architectural templates.

A closer examination of the GC10-DET dataset reveals the nuanced impact of backbone behavior under complex visual conditions. Here, the native YOLOv9-C configuration achieves the highest precision, despite several alternative backbones attaining higher recall and mAP/F1 scores. This outcome is strongly influenced by the dataset's inherent characteristics: GC10-DET features highly textured backgrounds and a predominance of sparse, small, and elongated defect types (e.g., Cr, In, Rp). Such conditions heighten the sensitivity of confidence calibration and Non-Maximum Suppression (NMS) to both proposal density and overlap. Under unified confidence and NMS settings, recall-oriented backbones tend to produce a larger volume of low-confidence proposals, which increases false positives in the presence of ambiguous background textures. In contrast, YOLOv9-C with its GELAN backbone applies more conservative confidence scoring and stronger suppression, effectively filtering out spurious detections and thereby achieving higher precision.

By comparison, the NEU-DET dataset with its more uniform imaging conditions and a smaller, more distinct set of defect classes shows different behavior. In this case, replacing the backbone tends to improve both recall and precision concurrently, and the native configuration's precision advantage becomes less pronounced. This contrast highlights that performance dynamics are not solely dependent on backbone architecture but are deeply coupled with dataset-specific factors such as image complexity, class granularity, and defect morphology. Thus, effective backbone selection requires considering both the visual nature of the data and the downstream calibration effects imposed by the detection framework.

To provide actionable insights for practitioners facing competing priorities whether optimizing for recall, F1 score, or computational efficiency, we present a controlled, cross-family evaluation. This includes six modern backbones tested under both YOLOv9-C and YOLOv5-m frameworks, with consistent training conditions including identical data splits, input resolution, augmentation, and schedules. Our deployment-oriented analysis further incorporates GFLOPs and batch-1 latency metrics, revealing that YOLOv9-C with RepViT offers the best overall mAP and F1, while FasterNet achieves the highest recall. In contrast, the same backbone families under YOLOv5-m consistently underperform at matched training budgets. These results reinforce a key finding: performance-compute trade-offs are not determined by the backbone alone, but rather by its interaction with the broader detection architecture and dataset-specific calibration effects–a relationship that has been largely overlooked in prior defect detection literature.

## 5 Conclusion

This study benchmarks diverse backbones for surface defect detection and articulates how recent advances in deep learning align with real world inspection requirements. The results provide a practical basis for selecting and optimizing backbones under industrial constraints and lay the groundwork for subsequent research on backbone optimization tailored to production settings. To understand failure modes, we analyze class-wise feature distributions and errors. Dataset visualization reveals a strong midline spatial prior and a predominance of small, extreme-aspect-ratio instances; crazing is particularly challenging due to tiny size, elongated geometry, and low local contrast, leading to near-miss IoU errors and NMS collisions. Our contribution is a reproducible selection framework rather than another detector variant: any future model (e.g., YOLOv10/YOLOv11 or transformer-based real-time detectors) can be plugged into the same protocol to extend the matrix. Despite the promising results, this study presents certain limitations that warrant further investigation. A key limitation is that persistent near-miss IoU errors and NMS collisions remain under challenging cases (tiny, low-contrast, extreme-aspect-ratio defects). Future work will:

- Broaden cross-dataset validation to assess how frequently these two error modes arise under different data distributions;
- Incorporate additional backbones and NMS-free paradigms to reduce suppression conflicts among densely packed, elongated instances;
- Explore oriented boxes/segmentation and threshold calibration within our unified protocol to improve localization around decision boundaries and mitigate close-call (near-miss) failures without changing the study's core conclusions.

Moreover, the evaluation was based exclusively on the NEU-DET and GC10-DET dataset, which may not adequately capture the diversity and complexity of surface defects encountered in real-world industrial environments. As a result, the generalizability of the proposed approach to more diverse or noise-prone scenarios remains to be verified. In addition, the dataset features relatively balanced defect categories, which contrasts with the class imbalance commonly observed in actual production settings. Future studies should explore defect detection under imbalanced class distributions and data-augmentation strategies to address this gap.

## Data Availability

Publicly available datasets were analyzed in this study. This data can be found at: http://faculty.neu.edu.cn/songkechen/zh_CN/zdylm/263270/list/.
